# An Investigation into the Role of P-Glycoprotein in the Intestinal Absorption of Repaglinide: Assessed by Everted Gut Sac and Caco-2 Cell Line

**Published:** 2019

**Authors:** Morteza Yaghoobian, Azadeh Haeri, Noushin Bolourchian, Soraya Shahhosseini, Simin Dadashzadeh

**Affiliations:** a *Department of Pharmaceutics, School of Pharmacy, Shahid Beheshti University of Medical Sciences, Tehran, Iran. *; b *Department of Medicinal Chemistry, School of Pharmacy, Shahid Beheshti University of Medical Sciences, Tehran, Iran. *; c *Pharmaceutical Sciences Research Center, Shahid Beheshti University of Medical Sciences, Tehran, Iran.*

**Keywords:** Repaglinide, Caco-2 Cells, Everted gut sac, P-glycoprotein, Permeability

## Abstract

The present study aimed at exploring the potential of the P-glycoprotein (P-gp) transporters as a barrier to the repaglinide (REG) epithelial permeability. *In-vitro* intestinal absorption models, the everted gut sac, and Caco-2 cell line, were used to study the possible role of P-gp in intestinal transport of REG. In the everted gut sacs, apparent permeability coefficients showed cargo concentration dependency transport over the concentration of 40 µM, indicating involvement of a saturable mechanism in REG absorption (P_app_ were 1.23 × 10 ^-5^ and 3.29 × 10 ^-5^ at drug concentrations of 40 and 100 μM, respectively). Adding verapamil (100 μM), valspodar (5 μM) and ketoconazole (10 μM) significantly enhanced the permeability of REG across mucosal to serosal in the rat jejunum (*P* < 0.05) suggesting role of CYP 3A4 and/or efflux transporters in oral bioavailability of REG. However, the results of Caco-2 cell experiments indicated low efflux ratios (less than 2) and insignificant involvement of P-gp efflux pumps in REG intestinal transport. Given that Caco-2 cells do not express adequate level of CYP 3A4, the current study suggests that the presystemic metabolism by cytochrome P450 (and not ejection by P-gp) may play a significant role in limiting the oral absorption of REG in small intestine.

## Introduction

Repaglinide (REG) is a fast-acting oral antihyperglycemic agent used for the treatment of non-insulin-dependent diabetes mellitus. It is particularly an attractive drug for diabetic patients with renal impairment, because it is mainly excreted through biliary route (1). When administered orally, REG is absorbed rapidly, but its bioavailabilty is low and variable (2). REG is primarily metabolized by hepatic enzymes cytochrome P450, particularly the CYP3A4 and CYP2C8 isoforms. Considering the high expression of CYP3A in both liver and intestine, the low and variable oral bioavailability of the drug may be caused by the intestinal or hepatic presystemic metabolism. However, it is not clear whether the low oral bioavailability of REG is the result of poor membrane transport or extensive presystemic metabolism. Intestinal absorption, which is usually influenced by various uptake and efflux membrane transporters in the intestine and/or liver, is an important key factor affecting the oral bioavailability of medications (3). P-gp is the most investigated transporter in the gut. This ATP binding cassette (ABC) efflux transporter is located on the apical membrane of the mature intestinal cells thereby limiting the intestinal absorption of many clinically important and frequently prescribed drugs (4, 5). Moreover, the joint presence of CYP3A and P-gp in enterocytes and the significant overlap in their substrates suggest their synergistic action in limiting oral drug absorption (6, 7). Due to the barrier role of P-gp in limiting the intestinal absorption of wide class of drugs, down-regulation, bypassing or inhibition of P-gp at the intestine has been considered as a strategy to improve oral drug bioavailability of known substrates. Despite the wide clinical use of REG, its interaction with P-gp has not been well investigated and the findings are controversial. Niemi *et al.* investigated possible associations between the pharmacokinetics of REG and single nucleotide polymorphisms (SNPs) in the genes encoding for the drug transporters organic anion transporting polypeptide 1B1 (OATP1B1) and P-gp (8). The results showed that pharmacokinetics of REG was not associated with the studied P-gp SNPs, suggesting that REG is not a substrate of P-gp. Based on their results OATP1B1 genotype was significantly associated with an enhanced effect of REG on blood glucose. However, according to some other studies, the P-gp is likely to affect REG and can significantly participate in drug-drug interactions with other P-gp substrates or inhibitors. Kajosaari *et al.* reported that coadministration of REG with cyclosporine A, significantly increased the plasma concentrations of REG in healthy volunteers. Inhibition of the CYP3A4- catalyzed metabolism as well as the OATP1B1-mediated hepatic uptake of REG by cyclosporine was considered as the probable cause of the observed interaction. It is of note that cyclosporine inhibits CYP3A4, P-glycoprotein, and OATP1B1 (9). Chang *et al.* by using pharmacophore models and database screening predicted the affinity of REG for P-gp and confirmed their prediction through inhibitory effects of the drug on [^3^H] digoxin transport in MDCK MDR1 *in-vitro* cell model (10). Considering the low and variable oral bioavailabilty of REG and controversy of reports regarding the involvement of P-gp transporter in REG oral absorption, the present study aimed at exploring the potential of the P-gp transporter as a barrier to the REG epithelial permeability. The permeability and transport characteristics of REG were investigated using the everted gut sac and Caco-2 cell models. 

## Experimental

*Materials*

Repaglinide (REG) was purchased from Darou Pakhsh Pharmaceutical Co. (Tehran, Iran). Roswell Park Memorial Institute-1640 (RPMI 1640), Dulbecco›s modified Eagle›s medium (DMEM), fetal bovine serum (FBS), penicillin-streptomycin solution and Hank’s balanced salt solution (HBSS) were obtained from Biowest (Germany). Valspodar was a generous gift from Novartis (Basel, Switzerland). Verapamil hydrochloride was supplied by Pars Darou (Tehran, Iran). HPLC grade acetonitrile and hydroxylethyl piperazine ethane sulfonic acid (HEPES) were obtained from Merck (Germany). Lucifer yellow was provided by Sigma-Aldrich (Germany). All other chemical reagents used were of analytical grade.

*Everted gut sac studies*

*Preparation of everted sacs*

The everted sac method was used as previously described (11). All animal experiments were approved by the local ethics committee of Shahid Beheshti University of Medical Sciences (Tehran, Iran). Male Wistar rats weighing 230 and 250 g were fasted for 24 h but were allowed free access to water. Under deep anesthesia, the jejunum of the rat intestines (approximately 10 cm) was quickly excised, and the underlying mesenterium was removed. The segment was washed several times with normal saline solution and placed immediately into the oxygenated Krebs buffer (composition: NaCl 118 mM, NaHCO_3_ 25 mM, KCl 4.7 mM, CaCl_2_2.5 mM, MgSO_4_ 1.2 mM, KH_2_PO_4_ 1.2 mM, and d-glucose 11 mM). The intestine was carefully everted on a glass rod (3.0 mm diameter), filled with 2 mL of Krebs buffer, and divided into sacs approximately 4.5 cm in length with silk suture. The sacs were placed in the oxygenated buffer solution (pH 6.8). The solution was maintained at 37 °C with 95% O_2_ and 5% CO_2_ throughout the experiment.

*Drug transport studies*

Sacs were placed in 25 mL of oxygenated buffer solutions (pH 6.8) containing different concentrations of REG (10 to 100 µM) with or without the P-gp inhibitors at the typically used inhibitory concentrations: verapamil (100 µM), valspodar (5 µM), or ketoconazole (10 µM) (12-14). At various time points, 10, 20, 35, 45, 60, and 90 min, sacs were removed, blotted dry, and the serosal contents were collected. REG serosal concentration was determined by a validated HPLC method (Section 2.4). Each sac was weighed before and after the experiment to calculate the fluid volume inside the sac. 

*Evaluation of the viability of the everted gut sacs*

The viability and any possible damage of the gut was determined by measuring the release of the cytosolic enzyme lactic dehydrogenase (LDH), as an indicator of cell damage, and analyzing the glucose concentrations both in the mucosal and serosal sides (11, 15). The LDH activity was measured in the incubation media using LDH-P kit from Kimia Pajouhan (Tehran, Iran). The results were calculated as U/L/cm^2^ of sac area. Glucose concentration was measured by a biochemistry analyzer (Roche Diagnostics, Germany).

**Table 1:** **Apparent permeability coefficient (P**_app_ **) of REG, across everted gut sacs at various drug concentrations** **(**Mean ± SD, n **= 3)**

**Concentration (µM)**	**P**_app_ ** × 10** ^-5^ ** (cm/s)**
10	1.21 ± 0.14
20	1.16 ± 0.09
40	1.23 ± 0.19
100	3.29 ± 0.58**

** *P* < 0.01: significant difference when compared to the lower REG lower concentrations.

**Table 2:** P_app_ (cm/s) and efflux ratio calculated for the Caco-2 cell studied at different concentrations of REG in the presence or absence of verapamil or valspodar (mean ± SD, n = 3)

**Compound**	**P**_app_ ** × 10** ^-5^ ** (cm/s)**	**ER**
	**A→B B →A**	
REG (20 µM )	1.67 ± 0.04 1.07 ± 0.02	0.64 ± 0.02
REG (40 µM )	1.84 ± 0.12 1.19 ± 0.16	0.65 ± 0.05
REG (60 µM )	1.83 ± 0.04 1.46 ± 0.11	0.80 ± 0.07
REG (40 µM ) + Verapamil (100 µM)	1.96 ± 0.27 1.18 ± 0.2	0.61 ± 0.09
REG (40 µM ) + Valspodar (5 µM)	1.64 ± 0.05 1.12 ± 0.1	0.68 ± 0.07

The influence of concentration on the transport of REG across the Caco-2 cell monolayers is demonstrated at three concentrations (20, 40 and 60 µM) (Figure 2, a and b). No saturation was observed for the transport of REG in both directions (apical to basolateral and basolateral to apical) at the concentrations studied.

**Figure 1:**
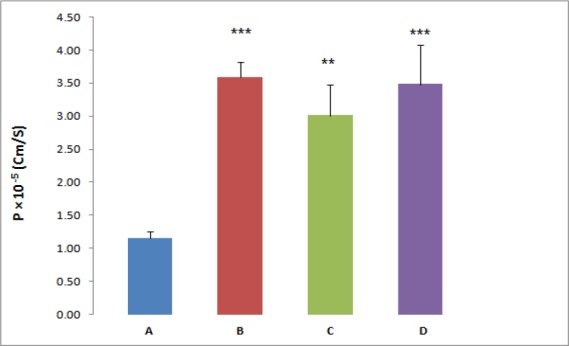
The effect of P-gp inhibitors on REG permeability across everted gut sacs, (A) REG 20 µM (Control), (B) REG plus 100 µM Verapamil, (C) REG plus 5 µM Valspodar, (D) REG plus 10 µM Ketoconazol,**P < 0.01, ***P < 0.001, significant difference compared to the control

**Figure 2:**
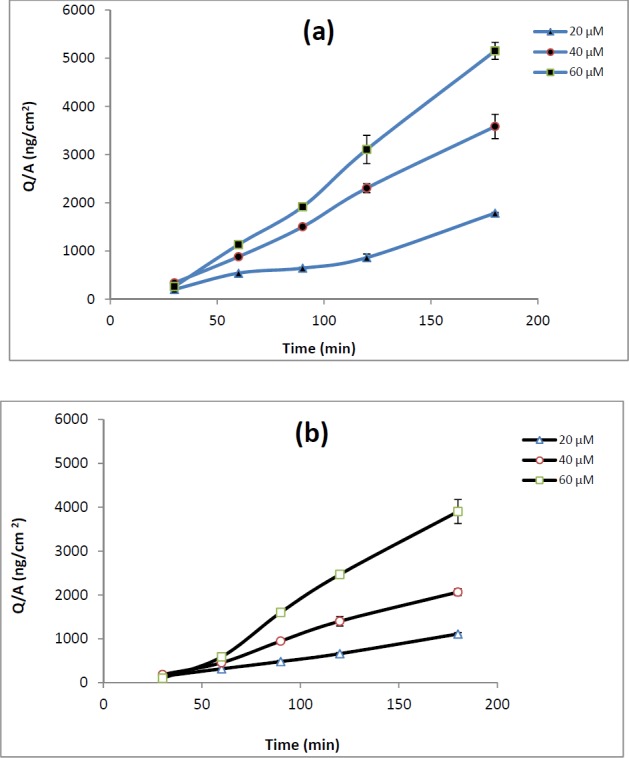
(a) Apical-to-basolateral permeation of REG 20 µM (), 40 µM () and 60 µM () in Caco-2 cell. (b) Basolateral-to-apical permeation of REG 20 µM (), 40 µM () and 60 µM () in Caco-2 cell

*Cell Culture studies*

Caco-2 cells (purchased from Pasteur Institute of Iran) were grown on 75 cm^2 ^plastic culture flasks, containing 15% (V/V) fetal bovine serum (FBS), 50% RPMI 1640, 34% DMEM and 1% (V/V) penicillin–streptomycin at 37 °C in a 95% humidified air with 5% CO_2_. The growth medium was changed every other day, and the cells were trypsinized using Trypsin-EDTA every 4 days for passage. The cells with passage numbers of 35-40 were used for the drug transport experiments.

*Transport studies*

When reaching 80–90% confluency, for transport study, the cells were seeded on polycarbonate filter inserts (with pore size of 0.4 µm, 1.13 cm^2^ surface area, Korea) at a density of 5 × 10^4 ^cells/cm^2 ^and grown for 21 days to allow full expression of P-gp and appropriate tight junctions. To evaluate the integrity of cellular monolayer, the transepithelial electrical resistance (TEER) was measured and transport studies were conducted when the TEER values were more than 300-400 Ωcm^2^. Before conducting drug transport experiments, the cells were washed twice with HBSS containing 25 mM HEPES (pH 7.4, 37 °C). Then, monolayers were pre-incubated at 37 °C for 30 min, and TEER values were measured. HBSS/HEPES buffer on both sides of the cell monolayers was then removed by aspiration and replaced with REG solution with various concentrations (20, 40 and 60 µM) on one side of the cell layer, apical (A) or basolateral (B), and fresh blank buffer on the other side. The volume of the buffer on the apical and basolateral side of the cell layer was 0.3 mL and 0.7 mL, respectively. The monolayers were incubated at 37 °C, placed in a shaker and shaken at 50 rpm during the transport process. The samples were taken from the receptor chamber (apical side or basolateral side in the case of B-A and A-B transport studies, respectively) at 30, 60, 90, 120 and 180 min, and then replaced with the same volume of the pre-warmed fresh buffer. Each experiment was repeated three times and REG concentration was determined by the HPLC system. The inhibition of REG efflux across Caco-2 cells was examined in the presence of two common P-gp inhibitors: verapamil (100 μM) and valspodar (5 μM).

*Integrity of the monolayer*

To determine the cell monolayers integrity, the TEER values and percent of Lucifer yellow transport across the cell monolayers were measured. The TEER was measured using an EVOM 2 Epithelial Voltohmmeter (World Precision Instrument, Sarasota, USA). The transport studies were conducted when the TEER values were more than 300-400 Ωcm^2^. A reduction in the TEER recorded across a monolayer indicates the dilation of tight junctions or a disruption of the monolayer (16, 17). Lucifer yellow is a small hydrophilic fluorescent molecule that is easily detectable by spectrophotofluorometer (485 nm excitation and 535 nm emission). The paracellular transfer of Lucifer yellow was determined and served as an additional marker for the epithelial cell tightness. In each set of experiments, Lucifer yellow was added (100 µg/mL) to the inserts and its passage was evaluated at 37 °C (18).

*Analysis of the samples*

HPLC was conducted using a system consisting of a Wellchrom K-1001 HPLC pump and a K-2700 diode array UV/VIS spectrophotometric detector (Knauer, Germany). The analyses were conducted at ambient temperature on a Perfect Sil RP-18 (150 mm × 4.6 mm *i.d.*, Merck). The optimal mobile phase for REG was a mixture of acetonitrile and phosphate buffer 0.02 M (60:40 V/V) delivered at a flow rate of 1 mL/min. The detection wavelength was set at 242 nm and the injection volume was 100 μL. The column temperature was maintained at room temperature. The calibration curves were linear over the concentration range of 100-1000 ng/mL (R^2 ^> 0.994). The intra-day and inter-day coefficients of the variation (CV) were all less than 5%.

*Calculations*

Apparent permeability coefficients (P_app_) of REG were calculated in everted gut sac and Caco-2 cell line according to the below equation (19):

P_app_ = (dQ/dt)/(A × C_0_)

Where the dQ/dt (ng/min) is drug permeation rate, A (cm^2^) the cross sectional area, and C_0_ (ng/mL) the initial concentration of REG in the donor chamber. In Caco-2 cell, the P_app_ of REG was calculated in both apical to basolateral (p_app.AB_), and basolateral to apical (p_app.BA_) directions. The efflux ratio (ER) of the drug in Caco-2 cell was assessed by calculating the ratio of p_app.BA _to p_app.AB_ (p_app.BA_*/*p_app.AB_). TEER was calculated based on the following equation (16):

TEER = (TEER_mono_ – TEER_blank_) × A 

Where TEER_mono_ is the cell monolayer and porous membrane resistance, TEER_blank_ the porous membrane resistance, and A the porous membrane surface area. The Lucifer yellow transport was calculated according to the following equation (18):

Lucifer Yellow Passage% = [(F_t _− F_b_)/(F_0 _− F_b_)] × 100

F_t _is the fluorescence intensity of Lucifer yellow across the monolayer, F_0_ the initial fluorescence intensity of Lucifer yellow on the donor side, and F_b_ the fluorescence intensity of the blank sample (HBSS/HEPES buffer alone).

*Statistical analysis*

The results are presented as mean ± standard deviation and analyzed using one-way ANOVA. Statistical significance level was set at *P < *0*.*05. 

## Results

*Absorptive transport of REG across everted gut sacs*

LDH is an intracellular enzyme which has been used as a biochemical marker of intestinal wall damage (20). In order to evaluate the viability of gut sac and any possible damage due to the sac preparation, the release of the cytosolic enzyme LDH was examined. At 30 min, the mean LDH activity detected in the incubation media was 150 U/L/cm^2^ and was similar to that detected at 60 and 90 min (*p* > 0.05), indicating the viability of the gut sacs during the experiments. Glucose is actively transported in the small intestine, thus a glucose gradient between the outside and inside fluids of gut sacs is considered as an additional indicator for gut viability and well functionality. In our experiments at 90 min, the glucose concentration in the serosal fluid was approximately 1.5 times higher than the mucosal concentration (data not shown). These results show that the sacs were biochemically active and physically intact during the experiments.

The transport of REG from mucosal (bulk solution) to serosal side (inside sac) of the jejunum at various REG concentrations (10, 20, 40 and 100 µM) was investigated. As shown in Table 1 there was no significant difference between the apparent absorptive permeability (P_app_) values when drug concentration was ≤40 µM, however an about 2.8-fold increase in the permeability value was observed at higher drug concentration (100 µM). The concentration dependent absorption of REG may reflect possible involvement of a saturable mechanism for its intestinal transport, either CYP3A enzymes, which are responsible for the drug intestinal first pass metabolism, or efflux transporters.

For further investigation, the effect of three different P-gp inhibitors (verapamil, valspodar and ketoconazole) on the REG transport across the everted gut sacs was examined; separately. According to the results the P_app _of REG was significantly affected by the employed inhibitors. In the presence of the P-gp inhibitor, verapamil (100 µM), valspodar (5 µM) or ketoconazole (10 µM), a marked increase in the absorptive flux of REG was observed (Figure 1). In the presence of verapamil, valspodar and ketoconazole and at REG concentration of 20 µM, a 3-fold, 2.6-fold and 3-fold increase in the drug permeability was observed, respectively (*P* < 0.01).

*Transport of repaglinide across Caco-2 Cells*

The involvement of P-gp in REG transport was evaluated by measuring bi-directional transport of drug across Caco-2 Cells. Monolayers displaying TEER values greater than 300 Ωcm^2^ were used for transport studies. Lack of significant difference between the TEER values before and after the experiments indicates the integrity of the monolayers during all the experiments. The integrity of the monolayer was additionally confirmed by monitoring the transport of Lucifer yellow, which is commonly used as a paracellular marker. The transport of Lucifer yellow was determined to be <0.3% in all the experiments. The P_app_ values of REG at apical to basolateral, p_app.AB_, and basolateral to apical, p_app.BA_, direction are shown in Table 2. 

The p_app.AB_ of REG did not show any significant changes over the studied concentrations. The efflux ratio remained almost constant (about 0.6) across the tested concentrations. As demonstrated in Table 2, adding verapamil (100 µM) or valspodar (5 µM) to the medium of Caco-2 cell monolayers did not affect drug transport or efflux ratio.

## Discussion

P-gp, a broad-spectrum efflux transporter, has a major impact on the pharmacokinetic behavior of most clinically relevant drugs. It is expressed in various tissues, particularly epithelial cells of gastrointestinal (GI) tract, liver, and kidney resulting in reduced oral absorption, limited distribution, and enhanced elimination of drugs (21, 22). A few studies have shown evidence that REG may be a P-gp substrate but the role of P-gp in REG transport remains controversial (23). Therefore, the present study aimed at assessing the role of P-gp in the transport of the REG by using Caco-2 cells and everted gut sacs. The everted gut sac model closely simulate the intestinal conditions and has been extensively employed in different steps of drug development studies such as multidrug resistance, drug interactions, and the impact of efflux transport modulators on the absorption of drugs. The advantages of this model include being simple in handling and the presence of a mucus layer which mimic GI condition (24). Likewise, the Caco-2 cell model is a well-characterized intestinal *in-vitro* model and good correlations between the Caco-2 data and the human *in-vivo* absorption results have been shown for various drugs (25, 26). In everted gut sac, the P_app_ of REG at the higher drug concentration (100 µM) was 2.8 fold greater than those observed at lower concentrations. This marked concentration dependent absorption implies that some sort of efflux transporters or capacity limited intestinal first pass metabolism may be involved in the REG oral absorption. For more clarification, the gut sac experiments were repeated in the presence of known inhibitors of P-gp. As shown in Figure 1 the REG absorption was significantly affected by adding inhibitors, verapamil (100 µM), valspodar (5 µM) or ketoconazole (10 µM), separately. The tested inhibitors caused a 2.6 to 3-fold increase in the REG permeability (*P* < 0.01).

To confirm the gut sac results the bi-directional transport of REG across Caco-2 cells in the presence and absence of P-gp inhibitors (valspodar and verapamil) was also evaluated. Lack of significant difference between the TEER values before and after the experiments along with a low passage of Lucifer yellow (<0.3%) confirmed that the monolayer integrity was intact during the experiments. 

The Caco-2 cell model is generally considered as a standard method to determine preliminarily whether a compound is a P-gp substrate or not. According to the FDA guidance for industry, there is an involvement of a drug efflux transporter in Caco-2 cells if the efflux ratio is ≥2 and if a significant (>50%) reduction in the efflux ratio is observed in the presence of an inhibitor of the corresponding transporter (27). 

The Caco-2 cells transport data of REG are shown in Table 2. A relatively high apparent permeability coefficient for the apical to basolateral REG transport (1.84 × 10^-5 ^± 0.12 cm/s at 40 µM drug concentration) suggests that the drug can be effectively absorbed in the intestinal tract**. **As demonstrated in Table 2, it was found that the value of ER was less than 2 at all the concentrations tested. Moreover, when compared with REG alone, P-gp inhibitors did not significantly improve the apical to basolateral absorption of REG in Caco-2 monolayers (Table 2). Considering the FDA guidance and previous reports on recognition of compound as a P-gp substrate as well as considering the obtained Caco-2 results in the present study, REG cannot be considered as a distinct P-gp substrate. REG is rapidly absorbed from the gastrointestinal tract after oral administration, but it has a low and variable bioavailability (2). However, it is not clear whether the low and variable oral bioavailability of REG is the result of poor membrane transport or extensive presystemic metabolism. Since REG is primarily metabolized by hepatic enzymes cytochrome P450, particularly the CYP3A4 isoform, and the CYP3A4 is the major cytochrome P450 enzyme present in a high level in enterocytes, it is more likely that the low bioavailability of REG is mainly due to intestinal/hepatic presystemic metabolism rather than ejection by efflux transporters. Verapamil, valspodar and ketoconazole are known inhibitors of P-gp, but these compounds are also inhibitors of CYP450 enzymes. While, the tested inhibitors markedly enhanced the REG transport through everted gut sacs (Table 1), they did not show any significant effect on the drug transport across Caco-2 cells. Considering that Caco-2 cells express adequate levels of functional P-gp, but do not express CYP 3A4 enzymes (7), the increased permeability of REG in the presence of the employed inhibitors in the gut sacs experiments could be mainly attributed to inhibition of CYP 3A4 activity. Our suggestion is more confirmed by the ketoconazole data in everted sacs. Among the three tested compounds, ketoconazole which is the most potent inhibitor of CYP 3A4 (28) showed a relatively higher enhancing effect on the REG transport. These results are consistent with a report by Niemi *et al.* showing that clarithromycin, a CYP3A4 inhibitor, significantly increased the AUC_0–∞_ and C_max_ of REG and enhanced its blood glucose-lowering effect (29). As mentioned before P-gp substrates vary greatly in molecular weight, chemical structure and function, however, several studies have been carried out to understand the molecular properties required for transport of drugs by this transporter. Some research groups have suggested that a log p value of at least 2.92, an 18-atom-long/longer molecular axis, a molecular weight of less than 800, and the presence of at least one basic nitrogen atom are common characteristics of the P-gp-substrates (30). REG have a large number of these characteristics; however our data suggest that REG seems not to be a P-gp substrate. Further studies are needed to fully characterize the possible interaction of REG with efflux transporters or CYP enzymes located in the small intestinal epithelial cells.

## Conclusion

To address limited data and the controversy of the results regarding involvement of P-gp transporters in REG oral absorption, in this study, the absorptive characteristics and the efflux mechanisms of REG were investigated using the everted gut sac and Caco-2 cell models. Our data showed concentration-dependent apparent permeability of REG in the absorptive direction as well as enhanced permeability by verapamil, valspodar, and ketoconazole treatment in the everted gut sac model. In the Caco-2 studies, the REG permeability remained unchanged in the presence of verapamil and valspodar, the potent P-gp inhibitors. These data support the major role of presystemic metabolism and the insignificant involvement of P-gp efflux pumps in low oral bioavailability of REG. 
